# A real-world analysis of FDA Adverse Event Reporting System (FAERS) events for liposomal and conventional doxorubicins

**DOI:** 10.1038/s41598-024-55185-4

**Published:** 2024-03-01

**Authors:** Huiling Su, Jing Jia, Yuxiang Mao, Riran Zhu, Zhengjun Li

**Affiliations:** 1 Department of Dermatology, Qilu Hospital, Shandong University, Jinan, Shandong China; 2 Department of Pharmacy, West China Hospital, Sichuan University, Chengdu, Sichuan China; 3 Department of Pharmacy, Affiliated Hospital of Shandong University of Traditional Chinese Medicine, Jinan, Shandong China; 4 Medical School, Kunming University of Science and Technology, Kunming, Yunnan China

**Keywords:** Drug regulation, Drug safety, Cancer, Cancer

## Abstract

The clinical application of conventional doxorubicin (CDOX) was constrained by its side effects. Liposomal doxorubicin was developed to mitigate these limitations, showing improved toxicity profiles. However, the adverse events associated with liposomal doxorubicin and CDOX have not yet been comprehensively evaluated in clinical settings. The FAERS data from January 2004 to December 2022 were collected to analyze the adverse events of liposomal doxorubicin and CDOX. Disproportionate analysis and Bayesian analysis were employed to quantify this association. Our analysis incorporated 68,803 adverse event reports related to Doxil/Caelyx, Myocet and CDOX. The relative odds ratios (RORs, 95%CI) for febrile neutropenia associated with CDOX, Doxil/Caelyx, and Myocet were 42.45 (41.44; 43.48), 17.53 (16.02; 19.20), and 34.68 (26.63; 45.15) respectively. For cardiotoxicity, they were 38.87(36.41;41.49), 17.96 (14.10; 22.86), and 37.36 (19.34; 72.17). For Palmar-Plantar Erythrodysesthesia (PPE), the RORs were 6.16 (5.69; 6.68), 36.13 (32.60; 40.06), and 19.69 (11.59; 33.44). Regarding onset time, significant differences adverse events including neutropenia, PPE, pneumonia and malignant neoplasm progression. This study indicates that clinical monitoring for symptoms of cardiotoxicity of CDOX and Myocet, and PPE and interstitial lung disease of Doxil should be performed. Additionally, the onset time of febrile neutropenia, malignant neoplasm progression, and pneumonia associated with Doxil and Myocet merits particular attention. Continuous surveillance, risk evaluations, and additional comparative studies between liposomal doxorubicin and CDOX were recommended.

## Introduction

Doxorubicin (DOX) works by intercalating into the DNA and subsequently inhibiting topoisomerase-II-mediated DNA repair^1^. It was a major component of many chemotherapy treatment regimens in clinical use^2^. According to the National Cancer Institute(NCI, https://www.cancer.gov), Dox was approved to be used alone or with other drugs to treat:acute lymphoblastic leukemia (ALL), acute myeloid leukemia (AML), breast cancer, gastric (stomach) cancer, hodgkin lymphoma, neuroblastoma, non-Hodgkin lymphoma, non-small cell lung cancer, ovarian cancer, small cell lung cancer, soft tissue and bone sarcomas, thyroid cancer, transitional cell bladder cancer, and wilms tumor. However, CDOX, either used alone or in combination with other chemotherapy drugs^3^, induces dose-dependent cardiotoxicity^4^, hematopoietic toxicity, hepatotoxicity, nephrotoxicity, and central neurotoxicity^5,6^. As a result, nano-scale delivery systems have been developed to reverse drug resistance and enhance the efficacy of doxorubicin. Several liposomal doxorubicin products, including but not limited to Doxil/Caelyx, and Myocet, have been marketed. While pegylated liposomal (Doxil) and non-pegylated liposomal (Myocet) formulations have unquestionably reduced drug toxicity, they have also introduced new toxicity issues.

**Figure 1:**
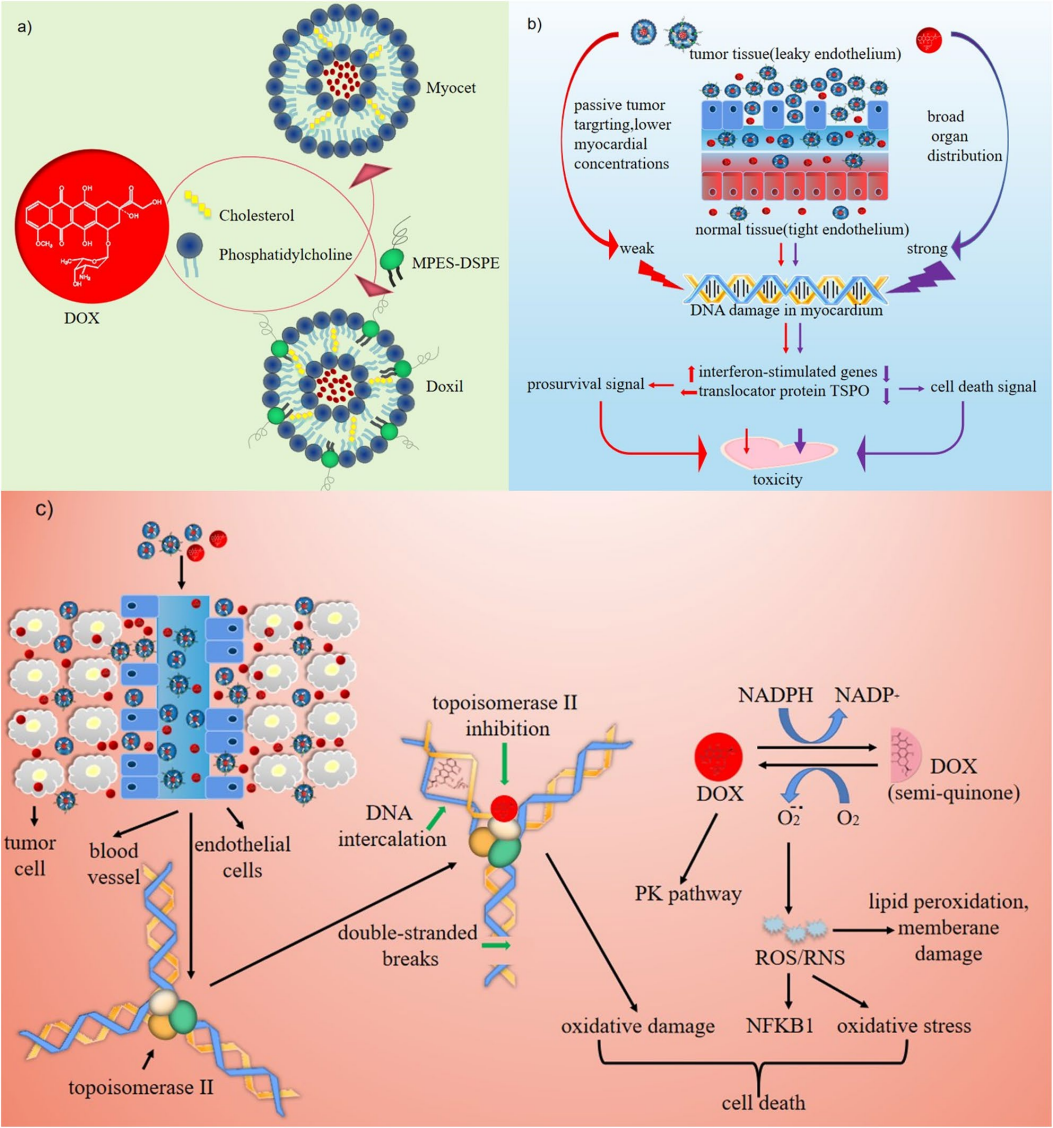
Drug nanocrystallization of DOX and tumor application. (**a**), nanocrystallization process of CDOX. (**b**), mechanisms of liposomal doxorubicin in enhancing permeation and retention and reducing cardiotoxicity. (**c**), mechanism of liposomal doxorubicin and CDOX inhibiting various solid tumors.

Doxil (USA) and Caelyx (Europe and Canada) were the same liposomal doxorubicin approved in different countries^7^. In 1995, Doxil received FDA approval for the treatment of various cancer types^8^. Doxil was a useful option in the treatment of various malignancies, including metastatic breast cancer^9^, ovarian cancer, multiple myeloma and AIDS-related kaposi's sarcoma^10^. Doxil was a liposomal formulation that modified surface properties using PEGylation based on CDOX^11^. This revolutionized the field of surface functionalization for liposomes^12^. This greatly reduced the risk of cardiotoxicity, no acute emesis, and lower alopecia, nausea^13^ or extravasation necrosis^14^ (Fig. 1). Numerous clinical research examined the therapeutic efficacy of Doxil in elderly patients with locally-advanced or metastatic breast cancer, with side effects including anaemia, mocusal inflammation^15^, PPE, infection and pulmonary embolism^16^, but no significant cardiotoxicity. The main toxicity of Doxil was mucocutaneous toxicity, such as PPE, also known as hand-foot syndrome^11,12^. Previous studies have demonstrated that this adverse reaction was caused by the toxic effect of polyethylene glycol-modified agent^15^. Myocet was a liposome-encapsulated formulation of the cytotoxic anthracycline doxorubicin, which differs from Doxil and CDOX^17^. Preclinical and clinical results showed that Myocet has similar drug efficacy and reduced PPE^12^ compared with Doxil^18–20^. Doxil significantly decreased cardiactoxicity and gastrointestinal toxicity^21^.

Meta-analyses of adverse events related to Doxil have primarily focused on side effects in ovarian cancer and breast cancer cardiotoxicity. In recurrent ovarian cancer, the toxicity profile of Doxil compared favorably with that of the comparator, despite PPE sometimes leading to treatment discontinuation. Doxil combined with carboplatin was associated with significantly more anemia and thrombocytopenia^22,23^. A meta-analysis of breast cancer based on 48 randomized controlled trials found that Doxil showed a trend towards lower cardiotoxic and cardiac event rates compared to CDOX^24–26^.

This study aims to use data mining to comprehensively evaluate and characterize the adverse events of liposomal doxorubicin compared to CDOX, using the Food and Drug Adverse Event Reporting System (FAERS) database. This will assist in establishing a set of evidence and expert consensus-based prevention and management recommendations.

## Results

### General characteristics

We analyzed a total of 68,803 (0.43%) adverse event reports associated with liposomal doxorubicin and conventional doxorubicin (CDOX), including 61,709 (89.69%) CDOX cases, 6663 (9.68%) Doxil cases, and 431 (0.63%) Myocet cases. The majority of the reports were submitted by physicians (42.63%) and other healthcare professionals (22.98%). The clinical characteristics of the events involving liposomal doxorubicin and CDOX were summarized in Table 1. The average onset age was slightly above 50 years and the mean weight was 59.6 kg, with more reports involving male than female patients (49.28% vs. 33.33%). The reports for CDOX and Doxil primarily came from the United States (31.50%, 39.80%), Canada (7.89%, 7.58%), and Germany (6.78%, 5.76%), while the reports for Myocet mainly originated from Germany (38.54%), Italy (17.20%), and Poland (11.15%). In terms of cancer prevalence, patients with malignant lymphoma (32.16%) showed the highest case rates, followed by breast cancer (10.56%). Notably, diffuse large B-cell lymphoma accounted for up to 40.91% of malignant lymphoma cases. The number of cases gradually increased over the years, with the largest increases observed between 2016 and 2019 (32.44%), followed by the period 2020–2022 (29.64%).

**Table 1:** Clinical characteristics.

Characteristics	CDOX	Doxil	Myocet	Total
Patient age, years				
Mean	49.24	57.37	54.64	50.14
< 18, n (%)	650 (1.77)	9 (0.21)	3 (0.96)	662 (1.60)
18–64, n (%)	3321 (9.05)	378 (8.96)	9 (2.87)	3708 (8.99)
≥ 65, n (%)	1174 (3.20)	181 (4.29)	14 (4.46)	1369 (3.32)
Unknown	31,570 (85.99)	3653 (86.54)	288 (91.72)	35,511 (86.09)
Wt, kg				
Mean	57.68	72.22	70.80	59.61
Reporting country, n (%)				
Austria	244 (0.66)	70 (1.66)	18 (5.73)	332 (0.80)
Canada	2895 (7.89)	320 (7.58)	(0)	3215 (7.79)
China	798 (2.17)	15 (0.36)	(0)	813 (1.97)
Germany	2490 (6.78)	243 (5.76)	121 (38.54)	2854 (6.92)
Spain	893 (2.43)	141 (3.34)	32 (10.19)	1066 (2.58)
France	2160 (5.88)	271 (6.42)	13 (4.14)	2444 (5.92)
United Kingdom	2169 (5.91)	168 (3.98)	6 (1.91)	2343 (5.68)
Italy	1791 (4.88)	204 (4.83)	54 (17.20)	2049 (4.97)
Japan	2033 (5.54)	183 (4.34)	2 (0.64)	2218 (5.38)
Poland	638 (1.74)	55 (1.30)	35 (11.15)	728 (1.76)
United states	11,564 (31.50)	1680 (39.80)	4 (1.27)	13,248 (32.12)
Other countries	9040 (24.62)	871 (20.63)	29 (9.24)	9940 (24.10)
Reporters, n (%)				
Consumer	3433 (9.35)	513 (12.15)	8 (2.55)	3954 (9.59)
Health-professional	4681 (12.75)	569 (13.48)	24 (7.64)	5274 (12.79)
Lawyer	266 (0.72)	228 (5.40)	(0)	494 (1.20)
Physician	15,609 (42.51)	1753 (41.53)	224 (71.34)	17,586 (42.63)
Other health-professional	8749 (23.83)	685 (16.23)	47 (14.97)	9481 (22.98)
Pharmacist	1626 (4.43)	237 (5.61)	10 (3.18)	1873 (4.54)
Unknown	2351 (6.40)	236 (5.59)	1 (0.32)	2588 (6.27)
Indications, n (%)				
Acute leukaemia	1747 (4.76)	66 (1.56)	17 (5.41)	1830 (4.44)
Acute lymphocytic leukaemia	1187 (67.95)	25 (37.88)	17 (100.00)	1229 (67.16)
Other acute leukaemia	560 (32.05)	41 (62.12)	(0)	601 (32.84)
Malignant lymphoma	12,448 (33.90)	755 (17.89)	63 (20.06)	13,266 (32.16)
Diffuse large B-cell lymphoma	4999 (40.16)	400 (52.98)	28 (44.44)	5427 (40.91)
B-cell lymphoma	1773 (14.24)	47 (6.23)	9 (14.29)	1829 (13.79)
Non-Hodgkin's lymphoma	1689 (13.57)	76 (10.07)	13 (20.63)	1778 (13.40)
Other lymphoma	3987 (32.03)	232 (30.72)	13 (20.64)	4232 (31.9)
Breast cancer	3787 (10.31)	400 (9.48)	170 (54.14)	4357 (10.56)
Lung cancer	89 (0.24)	11 (0.26)	1 (0.32)	101 (0.24)
Ovarian cancer	671 (1.83)	807 (19.12)	2 (0.64)	1480 (3.59)
Sarcoma	1943 (5.29)	161 (3.81)	(0)	2104 (5.10)
Osteosarcoma	345 (17.76)	19 (11.80)	(0)	364 (17.30)
Ewing's sarcoma	352 (18.12)	14 (8.70)	(0)	366 (17.40)
Kaposi's sarcoma	84 (4.32)	37 (22.98)	(0)	121 (5.75)
Other sarcoma	1162 (59.8)	91 (56.52)	(0)	1253 (59.55)
Nephroblastoma	86 (0.23)	3 (0.07)	(0)	89 (0.22)
Renal cancer	143 (0.39)	9 (0.21)	(0)	152 (0.37)
Multiple myeloma	1325 (3.61)	334 (7.91)	2(0.64)	1661 (4.03)
Other cancer/unkown indication	14,476 (39.43)	1675 (39.68)	59(3.64)	16,210 (7.03)
Reporting year, n (%)				
2004–2007	3042 (8.29)	577 (13.67)	4 (1.27)	3623 (8.78)
2008–2011	4006 (10.91)	502 (11.89)	33 (10.51)	4541 (11.01)
2012–2015	6721 (18.31)	626 (14.83)	134 (42.68)	7481 (18.14)
2016–2019	12,164 (33.13)	1143 (27.08)	73 (23.25)	13,380 (32.44)
2020–2022	10,782 (29.37)	1373 (32.53)	70 (22.29)	12,225 (29.64)
Sex, n (%)				
Female	17,331 (47.20)	2751 (65.17)	248 (78.98)	20,330 (49.28)
Male	12,724 (34.66)	993 (23.53)	33 (10.51)	13,750 (33.33)
Unknown	6660 (18.14)	477 (11.30)	33 (10.51)	7170 (17.38)
Total	36,715 (100.00)	4221 (100.00)	314 (100.00)	41,250 (100.00)

*CDOX* conventional doxorubicin, *Doxil* pegylated-liposome doxorubicin, *Myocet* non-pegylated-liposome doxorubicin, *n* the number of cases.

### Signal detection

Distinct risks of adverse events associated with liposomal doxorubicin and CDOX were identified (Table 2). Adverse events for liposomal doxorubicin and CDOX were analyzed using the reporting odds ratio (ROR), proportional reporting ratio (PRR), and Bayesian confidence propagation neural network (BCPNN). In total, 32 ROR signals were detected for all drugs, with CDOX, Doxil, and Myocet respectively accounting for 19, 18, and 11 positive signals. The results for the positive signals for liposomal doxorubicin and CDOX were summarized in Table 3.

**Table 2:** Signal detection.

SOC	Adverse events		Total	CDOX			Doxil			Myocet		
				n	ROR	95%CI	n	ROR	95%CI	n	ROR	95%CI
	PT code	PTs	68,803	61,709			6663			431		
Blood and lymphatic system disorders	10002034	Anaemia	4055	3609	4.24	4.10; 4.39	422	4.57	4.15; 5.04	24	4.01	2.67; 6.02
	10033661	Pancytopenia	2414	2166	9.36	8.96; 9.78	236	9.13	8.02; 10.39	12	7.13	4.03; 12.63
	10029354	Neutropenia	6991	6461	12.97	12.64; 13.31	474	8.18	7.45; 8.97	56	15.58	11.84; 20.49
	10043554	Thrombocytopenia	4041	3653	7.91	7.65; 8.18	360	7.00	6.30; 7.78	28	8.49	5.81; 12.40
Cardiac disorders	10007636	Cardiomyopathy	900	845	12.87	12.00; 13.81	53	7.05	5.38; 9.24	2	4.10	1.02; 16.44
	10048610	Cardiotoxicity	1192	1116	38.87	36.41; 41.49	67	17.96	14.10; 22.86	9	37.36	19.34; 72.17
Gastrointestinal disorders	10012735	Diarrhoea	3276	2972	1.06	1.02; 1.10	280	0.93	0.82; 1.05	24	1.25	0.83; 1.88
	10028813	Nausea	4180	3636	1.02	0.98; 1.05	533	1.41	1.29; 1.54	11	0.43	0.24; 0.79
	10047700	Vomiting	3284	2900	1.39	1.34; 1.45	372	1.68	1.51; 1.86	12	0.82	0.46; 1.46
General disorders and administration site conditions	10016288	Febrile neutropenia	9427	8868	42.45	41.44; 43.48	498	17.53	16.02; 19.20	61	34.68	26.63; 45.15
	10028116	Mucosal inflammation	2426	2214	21.68	20.74; 22.67	197	16.18	14.04; 18.65	15	19.02	11.40; 31.75
	10037660	Pyrexia	4997	4538	3.00	2.91; 3.09	405	2.45	2.22; 2.71	54	5.31	4.02; 7.01
Infections and infestations	10040047	Sepsis	3137	2940	6.05	5.82; 6.28	177	3.26	2.81; 3.78	20	5.79	3.71; 9.03
Metabolism and nutrition disorders	10059512	Apoptosis	6	6	4.30	1.91; 9.68	0	–	–	0	–	–
	10057248	Cell death	12	7	0.80	0.38; 1.68	5	5.35	2.22; 12.88	0	–	–
Musculoskeletal and connective tissue disorders	10003239	Arthralgia	854	754	0.42	0.39; 0.45	99	0.51	0.42; 0.63	1	0.08	0.01; 0.57
	10003988	Back pain	1018	819	0.76	0.71; 0.82	194	1.71	1.48; 1.97	5	0.67	0.28; 1.62
	10005956	Bone disorder	282	237	2.59	2.28; 2.95	44	4.47	3.32; 6.01	1	1.57	0.22; 11.16
	10006002	Bone pain	891	835	3.14	2.93; 3.36	52	1.79	1.37; 2.36	4	2.14	0.80; 5.72
	10031264	Osteonecrosis	593	526	3.06	2.81; 3.34	65	3.49	2.74; 4.46	2	1.66	0.41; 6.65
Neoplasms benign, malignant and unspecified (incl cysts and polyps)	10051398	Malignant neoplasm progression	2180	1795	4.36	4.16; 4.58	369	8.40	7.57; 9.32	16	5.56	3.38; 9.13
Nervous system disorders	10008190	Cerebrovascular accident	292	254	0.30	0.27; 0.34	34	0.38	0.27; 0.53	4	0.70	0.26; 1.86
	10019211	Headache	1249	1094	0.37	0.35; 0.39	151	0.48	0.41; 0.57	4	0.20	0.07; 0.52
	10024382	Leukoencephalopathy	153	139	8.19	6.91; 9.72	14	7.42	4.39; 12.55	0	–	–
	10033775	Paraesthesia	605	507	0.67	0.62; 0.73	93	1.16	0.94; 1.42	5	0.97	0.40; 2.33
	10044565	Tremor	257	223	0.28	0.25; 0.32	30	0.35	0.25; 0.50	4	0.73	0.27; 1.96
Psychiatric disorders	10002855	Anxiety	1614	1409	1.04	0.99; 1.10	204	1.42	1.23; 1.63	1	0.11	0.01; 0.75
Respiratory, thoracic and mediastinal disorders	10022611	Interstitial lung disease	1649	1355	6.81	6.44; 7.19	291	13.49	12.00; 15.17	3	2.09	0.67; 6.50
	10035598	Pleural effusion	1315	1148	4.12	3.88; 4.37	162	5.36	4.59; 6.27	5	2.54	1.05; 6.12
	10035664	Pneumonia	3214	2914	1.93	1.86; 2.00	278	1.70	1.51; 1.92	22	2.11	1.38; 3.22
Skin and subcutaneous tissue disorders	10033553	Palmar-plantar erythrodysaesthesia syndrome	1024	626	6.16	5.69; 6.68	384	36.14	32.60; 40.06	14	19.69	11.59; 33.44
Vascular disorders	10037377	Pulmonary embolism	1275	1143	2.48	2.34; 2.63	120	2.41	2.02; 2.89	12	3.77	2.13; 6.67

*SOC* system organ class, *n* the number of cases, *95% CI* 95%CI lower and 95% CI upper, *PT* preferred terms.

**Table 3:** Positive signal detection results of liposomal doxorubicin and CDOX.

SOC	PTs	CDOX			Doxil			Myocet		
		ROR (95%CI)	PRR (X^2^)	IC025	ROR (95%CI)	PRR (X^2^)	IC025	ROR (95%CI)	PRR (X^2^)	IC025
Blood and lymphatic system disorders	Anaemia	4.24 (4.10; 4.39)	4.12 (8402.09)	1.90	4.57 (4.15; 5.04)	4.42 (1125.53)	1.81	4.01 (2.67; 6.02)	3.89 (52.13)	0.47
	Pancytopenia	9.36 (8.96; 9.78)	9.17 (14,995.22)	2.98	9.13 (8.02; 10.39)	8.94 (1659.32)	2.68	7.13 (4.03; 12.63)	7.02 (62.09)	0.44
	Neutropenia	12.97 (12.64; 13.31)	12.14 (62,022.04)	3.42	8.18 (7.45; 8.97)	7.84 (2829.84)	2.64	15.58 (11.84; 20.49)	14.31 (697.06)	2.63
	Thrombocytopenia	7.91 (7.65; 8.18)	7.64 (20,281.80)	2.76	7.00 (6.30; 7.78)	6.79 (1778.29)	2.39	8.49 (5.81; 12.40)	8.16 (176.88)	1.47
Cardiac disorders	Cardiomyopathy	12.87 (12.00; 13.81)	12.76 (8528.89)	3.33	7.05 (5.38; 9.24)	7.02 (272.40)	1.76	–	–	–
	Cardiotoxicity	38.87 (36.41; 41.49)	38.41 (33,183.86)	4.73	17.96 (14.10; 22.86)	17.84 (1053.77)	3.03	37.36 (19.34; 72.17)	36.85 (313.55)	0.93
General disorders and administration site conditions	Febrile neutropenia	42.45 (41.44; 43.48)	38.51 (264,943.39)	4.90	17.53 (16.02; 19.20)	16.71 (7301.35)	3.70	34.68 (26.63; 45.15)	31.49 (1803.81)	3.53
	Mucosal inflammation	21.68 (20.74; 22.67)	21.19 (37,920.92)	4.09	16.18 (14.04; 18.65)	15.88 (2723.60)	3.40	19.02 (11.40; 31.75)	18.60 (249.98)	1.50
	Pyrexia	3.00 (2.91; 3.09)	2.90 (5662.74)	1.42	2.45 (2.22; 2.71)	2.39 (333.59)	0.92	5.31 (4.02; 7.01)	4.95 (172.95)	1.29
Infections and infestations	Sepsis	6.04 (5.82; 6.28)	5.89 (11,590.60)	2.39	3.26 (2.81; 3.78)	3.22 (271.75)	1.17	5.78 (3.71; 9.03)	5.64 (76.70)	0.76
Musculoskeletal and connective tissue disorders	Bone disorder	2.59 (2.28; 2.95)	2.59 (228.21)	0.92	4.47 (3.32; 6.01)	4.46 (117.72)	1.07	–	–	–
	Bone pain	3.14 (2.93; 3.36)	3.12 (1181.85)	1.39	–	–	–	–	–	–
	Osteonecrosis	3.06 (2.81; 3.34)	3.05 (712.26)	1.30	3.49 (2.74; 4.46)	3.48 (114.72)	0.94	–	–	–
Neoplasms benign, malignant and unspecified (incl cysts and polyps)	Malignant neoplasm progression	4.36 (4.16; 4.58)	4.30 (4453.89)	1.92	8.40 (7.57; 9.32)	8.13 (2305.32)	2.64	5.56 (3.38; 9.13)	5.44 (58.30)	0.51
Nervous system disorders	leukoencephalopathy	8.19 (6.91; 9.72)	8.18 (836.43)	2.34	7.42 (4.39; 12.55)	7.41 (77.29)	0.69	–	–	–
Respiratory, thoracic and mediastinal disorders	Interstitial lung disease	6.81 (6.44; 7.19)	6.72 (6361.84)	2.51	13.49 (12.00; 15.17)	13.13 (3241.89)	3.26	–	–	–
	Pleural effusion	4.12 (3.88; 4.37)	4.08 (2615.42)	1.80	5.36 (4.59; 6.27)	5.29 (563.81)	1.85	–	–	–
Skin and subcutaneous tissue disorders	Palmar-plantar erythrodysaesthesia syndrome	6.16 (5.69; 6.68)	6.13 (2596.88)	2.30	36.13 (32.60; 40.06)	34.78 (12,345.37)	4.63	19.69 (11.59; 33.44)	19.28 (242.77)	1.42
Vascular disorders	Pulmonary embolism	2.48 (2.34; 2.63)	2.46 (986.82)	1.09	2.41 (2.02; 2.89)	2.40 (98.07)	0.65	–	–	–

*ROR* reporting odds ratio, *PRR* proportional reporting ratio, *IC025* the lower limit of the 95% two-sided CI of the IC.

The RORs (95% CI) for febrile neutropenia associated with CDOX, Doxil, and Myocet were 42.45 (41.44; 43.48), 17.53 (16.02; 19.20), and 34.68 (26.63; 45.15) respectively. For cardiotoxicity, the RORs (95%CI) were 38.87 (36.41; 41.49), 17.96 (14.10; 22.86), and 37.36 (19.34; 72.17) respectively. The RORs (95% CI) for PPE were 6.16 (5.69; 6.68), 36.13 (32.60; 40.06), and 19.69 (11.59; 33.44) respectively. Additionally, the RORs (95% CI) for cardiomyopathy associated with CDOX and Doxil were 12.87 (12.00; 13.81) and 7.05 (5.38; 9.24), and for leukoencephalopathy associated with CDOX and Doxil were 8.19 (6.91; 9.72) and 7.42 (4.39; 12.55). The RORs (95% CI) for interstitial lung diseases with CDOX and Doxil were 6.81 (6.44; 7.19) and 13.49 (12.00; 15.17).

**Figure 2:**
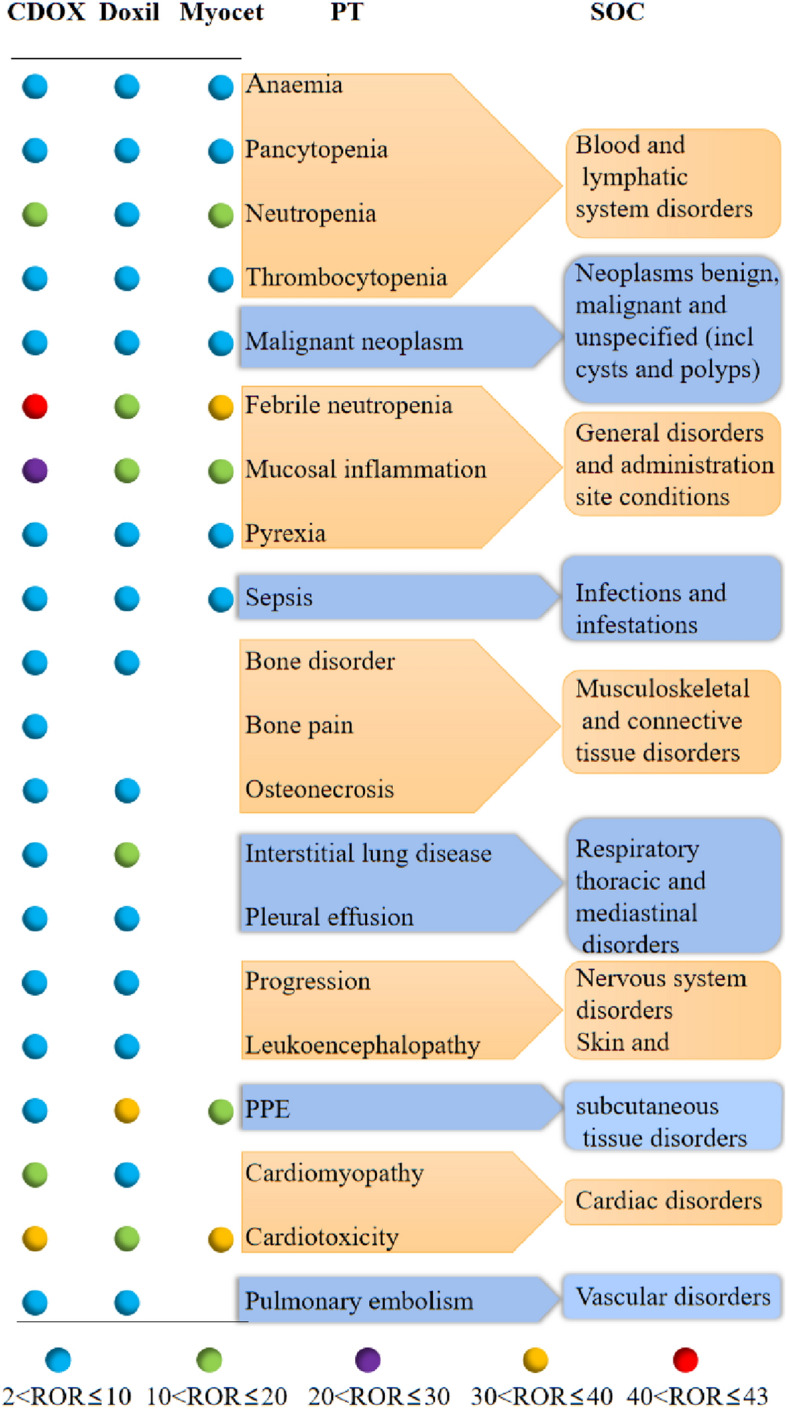
Summary of main adverse events of liposomal doxorubicin and CDOX.

For CDOX and Myocet, the RORs for cardiotoxicity and febrile neutropenia ranked highly. For Doxil, the ROR for PPE ranked highly. Analysis of the FAERS data suggests that the RORs for cardiomyopathy, febrile neutropenia, mucosal inflammation, and pyohemia associated with the use of Doxil were lower than those associated with CDOX. However, the RORs for PPE and interstitial lung diseases associated with Doxil treatment were higher than those associated with CDOX. The primary adverse events of liposomal doxorubicin and CDOX were summarized in Fig. 2.

### Onset time of events

In total, 13 cases reported the onset time. Upon comparing liposomal doxorubicin and CDOX, we found significant differences in 5 adverse events, namely neutropenia, palmar-plantar erythrodysesthesia (PPE), pneumonia, pulmonary embolism, and malignant neoplasm progression (Table 4). Myocet was excluded from the discussion for malignant neoplasm progression as the number of cases was less than 2.

**Table 4:** Onset time of events (days).

SOC	PTs	CDOX			Doxil			Myocet		
		n	Median (IQR)	Mean	n	Median (IQR)	Mean	n	Median (IQR)	Mean
Blood and lymphatic system disorders	Neutropenia	**75**	**35 (14; 273)**	**90.85**	**59**	**17 (10; 47)**	**66.35**	**25**	**30 (11.5; 78.5)**	**79.68**
	Pancytopenia	44	58.5(15; 123)	57.89	61	50 (15; 133.5)	55.06	6	80.5 (6.5; 121.5)	51.75
	Thrombocytopenia	32	14 (8; 91)	39.67	52	45 (14.25; 102)	53.74	11	16 (14; 59)	45.09
	Febrile neutropenia	124	33 (11; 77.25)	123.37	92	34 (12; 80.5)	128.23	29	12 (9; 86.5)	104.83
Cardiac disorders	Cardiomyopathy	24	126 (89; 325.5)	19.79	16	230 (121.5; 252)	21.56	1	–	–
General disorders and administration site conditions	Mucosal inflammation	24	15 (8; 29)	22.77	16	11 (6.25; 66)	23.41	7	20 (14; 50)	29.57
Infections and infestations	Sepsis	19	24 (9; 101)	24.82	26	51.5 (17.75; 107.5)	31.4	11	61 (10; 61)	28
Neoplasms benign, malignant and unspecified (incl cysts and polyps)	Malignant neoplasm progression	**11**	**427 (153; 1371)**	**52.41**	**63**	**142 (56; 445)**	**34.9**	1	–	–
Respiratory, thoracic and mediastinal disorders	Interstitial lung disease	24	105 (51.5; 114)	37.94	58	99 (42; 120.25)	46.33	3	49 (9; .)	19.17
	Pneumonia	**43**	**159 (16; 365)**	**52.15**	**37**	**17 (4; 290)**	**35.91**	**9**	**90 (41;116)**	**48.22**
	Pleural effusion	12	62 (20.75; 250)	22.75	28	79 (19.25; 177.5)	19.54	1	–	–
Skin and subcutaneous tissue disorders	Palmar-plantar erythrodysaesthesia syndrome	**11**	**60 (30; 72)**	**46.86**	**74**	**46 (30; 67.75)**	**45.28**	**10**	**72 (56.5;96.75)**	**69.4**
Vascular disorders	Pulmonary embolism	**16**	**96.5 (86; 1611)**	**29.84**	**25**	**49 (15.5; 95)**	**19.46**	**6**	**85 (49.5;200.25)**	**27.33**

*n* number of cases with available time-to-onset, *IQR* interquartile range, *Mean* rank average, *CI* confidence interval, *IC* information component, *ROR* reporting odds ratio, *PRR* proportional reporting ratio, *IC025* the lower limit of the 95%two-sided CI of the IC, bold said statistical results have significant difference, *p* < 0.05.

The median (IQR) for neutropenia associated with CDOX, Doxil, and Myocet were 35 (14; 273), 17 (10; 47), and 30 (11.5; 78.5), respectively. For PPE, the median (IQR) was 60 (30; 72), 46 (30; 67.75), and 72 (56.5; 96.75). For pneumonia, the median (IQR) was 159 (16; 365), 17 (4; 290), and 90 (41; 116). For pulmonary embolism, the median (IQR) was 96.5 (86; 1611), 49 (15.5; 95), and 85 (49.5; 200.25).

**Figure 3:**
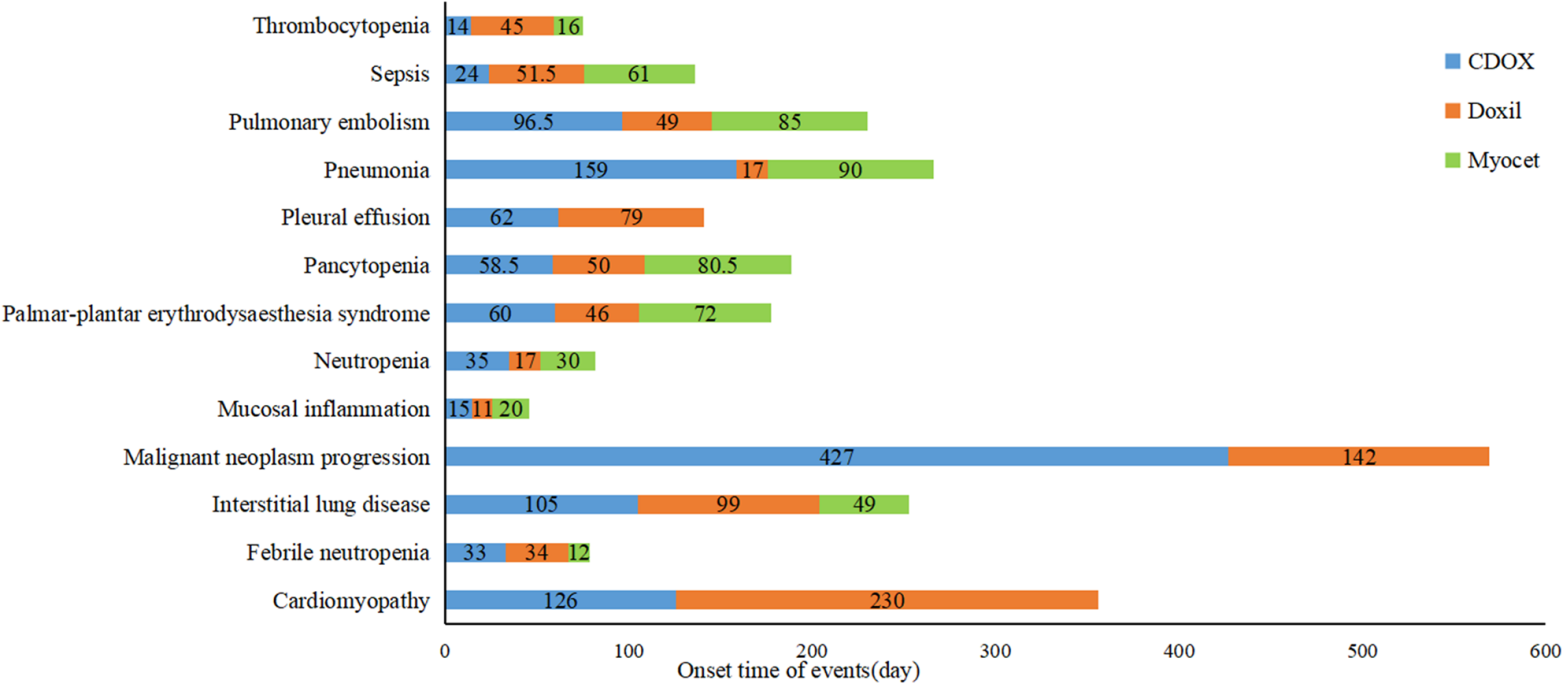
The onset time chart of liposomal doxorubicin and CDOX.

Mucosal inflammation had the shortest onset time, while malignant neoplasm progression had the longest. The onset times for malignant neoplasm progression and pneumonia for Doxil were significantly shorter than those for CDOX. Additionally, the onset times for cardiomyopathy, sepsis, and thrombocytopenia were longer for Doxil compared to CDOX. Conversely, for Myocet, the onset times for PPE, pancytopenia, and sepsis were longer than those for CDOX, while the onset time for febrile neutropenia was shorter than for CDOX (Fig. 3).

### Outcome events

**Figure 4:**
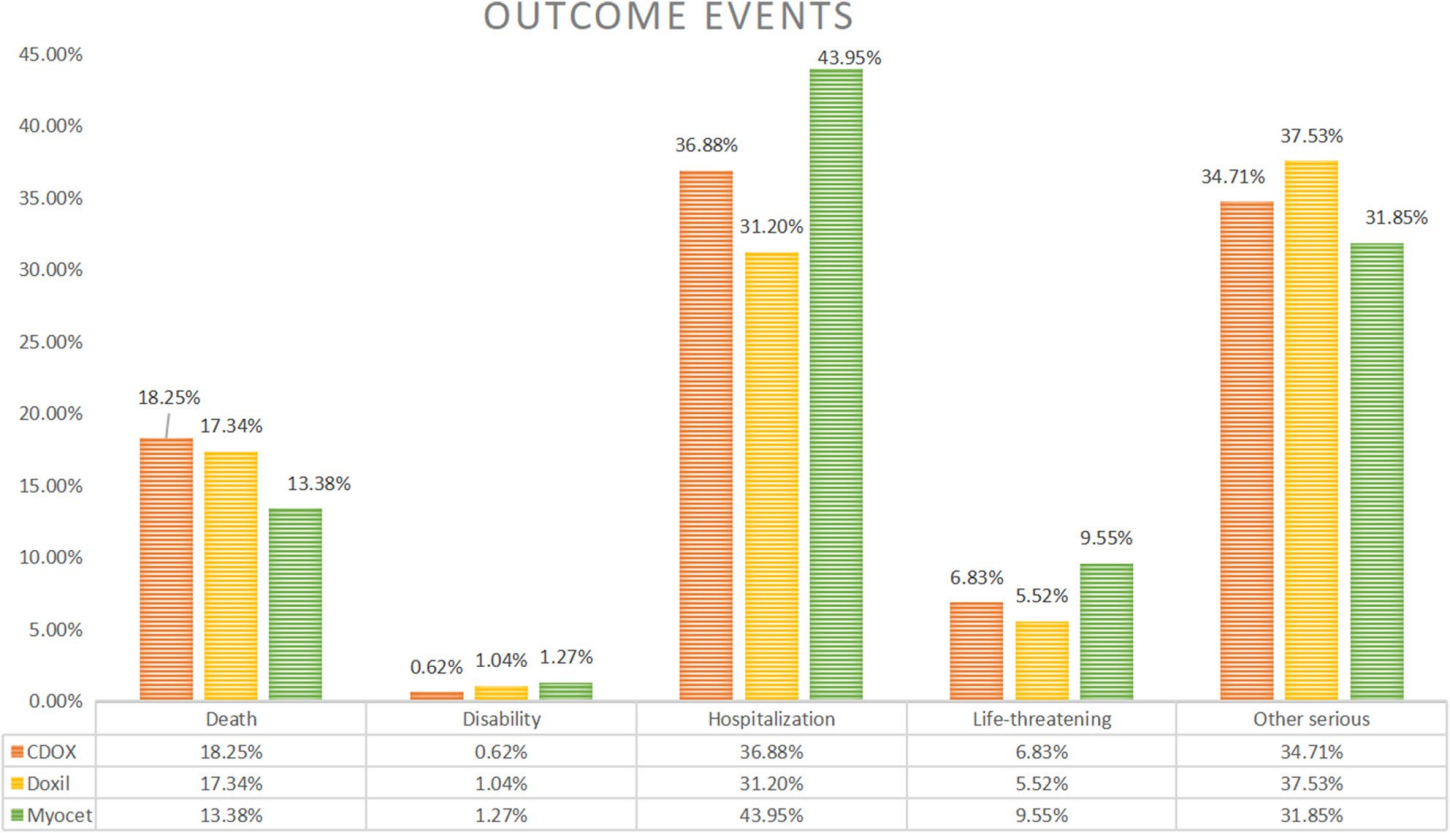
The outcome events chart of liposomal doxorubicin and CDOX.

We evaluated the outcomes reported to determine the prognosis of patients who experienced adverse events following liposomal doxorubicin and CDOX treatments (Table 5). Among all adverse events, the lowest risk was for disability (0.62%, 1.04%, and 1.27%), while the highest was for initial or prolonged hospitalization (36.88%, 31.20%, and 43.95%). The highest proportion of fatalities occurred in CDOX patients, followed by Doxil (17.34%), with the lowest observed in Myocet patients (13.38%) (Fig. 4).

**Table 5:** Outcome events.

	CDOX	Doxil	Myocet
Total reports	36,715	4221	314
Outcome events, n (%)			
Death	6700 (18.25)	732 (17.34)	42 (13.38)
Disability	226 (0.62)	44 (1.04)	4 (1.27)
Hospitalization-initial or prolonged	13,542 (36.88)	1317 (31.20)	138 (43.95)
Life-threatening	2507 (6.83)	233 (5.52)	30 (9.55)
Other serious (important medical event)	12,742 (34.71)	1584 (37.53)	100 (31.85)
Unknown	998 (2.72)	311 (7.36)	(0)

## Discussion

This study provides an updated, comprehensive analysis of the characteristics and differences in adverse events following treatment with liposomal doxorubicin compared to CDOX. Due to its enhanced permeability, retention, and lower cardiotoxicity, Doxil plays a significant role in the clinical setting. The majority of preferred terms (PTs) were chosen based on previous reports by Fukuda et al.^7^, thus complementing findings that were previously unattainable due to a lack of cases. Despite its benefits, liposomal doxorubicin can cause additional discomfort to patients, thereby reducing their quality of life. Consequently, a comprehensive disproportionality analysis for liposomal doxorubicin and CDOX was crucial for optimal clinical application and management.

Regarding the study of adverse events of CDOX and liposomal doxorubicin, our results were different from those of Fukuda et al. We identified several significant differences. Overall, the response to adverse events was better with liposomal DOX and CDOX, and several key findings emerged that warrant further discussion.

Randomized controlled trials have demonstrated that Doxil exhibits comparable anti-tumor activity and significantly reduced cardiotoxicity compared to CDOX in multiple breast cancer patients^13,27^. PPE, frequently observed in Doxil usage, was not seen with Myocet^21^. However, our results showed that the ROR for PPE was noticeably higher with Doxil compared to CDOX. The ROR for PPE in patients receiving Myocet was also high and could potentially be closely related to the dose or duration of Myocet treatment^28,29^. A retrospective trial of Myocet in lymphoma patients revealed that the most common grade 3/4 toxicity was hematological, including leukopenia, neutropenia, thrombocytopenia, and febrile neutropenia. In contrast, the main toxicity of Doxil was mucocutaneous^27,30^. Our study showed that the reporting odds ratios (RORs) for cardiotoxicity and febrile neutropenia were significantly lower for Doxil, compared to CDOX. However, Myocet did not appear to reduce the risk of cardiotoxicity and febrile neutropenia as effectively. Fukuda et al. suggested that Doxil and Myocet had lower RORs for cardiotoxicity and higher RORs for PPE compared to CDOX^7^. Our results of Doxil were largely consistent with Fukuda, and the PPE deserved attention when using Doxil. Interestingly, our results on Myocet in terms of cardiotoxicity were diametrically opposed to those of Fukuda et al. reported fewer than two cases of cardiotoxicity for Myocet, while the RORs for cardiotoxicity were considered low for Myocet. Our data suggested that Myocet appeared to have little advantage over CDOX. In the retrospective and prospective study of Sancho et al., it was found that using Myocet instead of CDOX was not associated with reduced early cardiotoxicity, although some reduced cardiac safety signals were observed^31^. Our results were consistent with that of Sancho et al. Age was one of the risk factors for cardiotoxicity. This may be related to the number of cases and the patient's age.

In our analysis, the age of most patients was concentrated between 18 and 64, with a mean onset age just over 50 years and a mean weight of 59.6 kg. Over the past 19 years, the number of reported cases has steadily increased. It was reported that the first exposure to Doxil can lead to immediate hypersensitivity reactions (HSRs)^32^. The symptoms include dyspnea, tachypnea, facial flushing, facial swelling, headache, hypertension or hypotension, chills, chest pain and back pain^32^. We have analyzed the adverse events of headache, back pain and paraesthesia caused by liposomal doxorubicin and CDOX, but they did not show significant differences in these adverse events. First of all, it may be that this adverse reaction can be pre-treated with corticosteroids and antihistamines^32^. Secondly, most patients with an initial reaction can finish the first infusion at a slower infusion rate after interruption and recovery, and usually tolerate further infusions^32^. Finally, the occurrence of HSRs depends on Doxil with high doses or short dosing intervals^29^. Because the FAERS database does not count dosages of Doxil used by patients, we were unable to collect data. Therefore, the HSRs of Doxil cannot be accurately interpreted.

The indications of DOX in this paper include approved and off-label cancer types. According to the National Cancer Institute (NCI, https://www.cancer.gov), Dox was approved to be used alone or with other drugs to treat: acute lymphoblastic leukemia (ALL), acute myeloid leukemia (AML), breast cancer, gastric (stomach) cancer, hodgkin lymphoma, neuroblastoma, non-Hodgkin lymphoma, non-small cell lung cancer, ovarian cancer, small cell lung cancer, soft tissue and bone sarcomas, thyroid cancer, transitional cell bladder cancer, and wilms tumor. In 1995, Doxil received FDA approval for the treatment of various disease types^8^ including Ovarian cancer^33^, Multiple myeloma, and AIDS-related Kaposi sarcoma. In addition, there were many clinical trials and literatures supporting Doxil for breast cancer^9^, lung cancer^34^, soft tissue and bone sarcoma^35^, renal cancer^36^, prostate cancer^37^, and cervical squamous cell carcinoma^38^.

In terms of cancer prevalence, patients with malignant lymphoma exhibited the highest reporting rate, especially those with diffuse large B-cell lymphoma. The combination of Doxil and carboplatin resulted in a higher incidence but shorter duration of mucosal perfusion and PPE^30^. In our study, the onset time for mucosal inflammation after treatment with liposomal doxorubicin and CDOX was the shortest, while that for malignant neoplasm progression was the longest. The onset times for cardiomyopathy, sepsis, and thrombocytopenia were longer for Doxil compared to CDOX. Moreover, the onset times for PPE, pancytopenia, and sepsis were longer for Myocet compared to CDOX. The onset time of PPE for Myocet was longer than that for CDOX, whereas the onset time for Doxil was shorter. This suggests that Doxil more readily and quickly induces mucosal inflammation and PPE. Additionally, the onset times of malignant neoplasm progression and lung disease for Doxil were shorter than for CDOX, indicating these conditions were more easily triggered by Doxil.

The Myocet did not reduce cardiotoxicity while maintaining efficacy and appeared to have no advantage. Compared to CDOX, Doxil shows equivalent antitumor activity with fewer side effects, but further strategies were needed to mitigate these adverse reactions. Continuous monitoring, risk evaluations, and additional comparative studies of liposomal doxorubicin and CDOX should be considered. This will provide a reference for the safe and effective clinical use of liposomal doxorubicin and CDOX.

Due to inherent limitations of clinical trials, such as patients having multiple disease types, some patients experiencing several adverse events simultaneously, the relatively small sample size for certain adverse events, differences in the occupation and professional levels of the reporters, non-standardized time recording, and the possibility of false positive signals in the signal detection method, we may not be able to fully unravel the intricacies of this study. Thus, further clinical trials were required to inform drug selection in clinical practice.

## Conclusions

Liposomal doxorubicin and CDOX must be applied with caution. Our analysis of FAERS data indicates that the RORs for cardiomyopathy, febrile neutropenia, mucosal inflammation, and pyohemia resulting from Doxil use were lower than those associated with CDOX. Moreover, the ROR for PPE was higher in patients treated with Doxil than those treated with Myocet, and the ROR for interstitial lung diseases was higher in patients treated with Doxil than those treated with CDOX. The RORs for cardiotoxicity, febrile neutropenia, pyrexia, and neutropenia were higher in patients treated with Myocet than those treated with Doxil.

We found that the onset time for malignant neoplasm progression and pneumonia was significantly shorter with Doxil than with CDOX through examining the onset time of adverse events. Conversely, the onset times for cardiomyopathy, sepsis, and thrombocytopenia were longer for Doxil than for CDOX. In the case of Myocet, the onset times for PPE, pancytopenia, and sepsis were longer than those for CDOX and Doxil, while the onset time for febrile neutropenia was shorter. In evaluating the outcome of events, liposomal doxorubicin and CDOX presented a lower risk of disability as an adverse event. However, the risk of initial or prolonged hospitalization was the highest, followed by other serious adverse events. The death rate for patients using CDOX and Doxil was slightly higher than for those using Myocet, although the opposite was found in life-threatening reports.

The results of this study show that cardiotoxicity and febrile neutropenia should be carefully monitored when using CDOX and Myocet. In the case of Doxil, it was particularly necessary to monitor PPE and interstitial lung disease. Additionally, the onset time of febrile neutropenia, malignant neoplasm progression, and pneumonia caused by liposomal doxorubicin requires careful attention. Continuous monitoring, risk assessments, and further comparative studies of liposomal doxorubicin and CDOX should be considered.

## Materials and methods

Data source. The FAERS database (https://fis.fda.gov/extensions/FPD-QDE-FAERS/FPD-QDE-FAERS.html) encompasses demographic and administrative details, drug information, Medical Dictionary for Regulatory Activities’ (MedDRA) preferred terminology (PT) for adverse event (REAC) coding, patient outcomes, reporting sources, initiation and end dates of treatment reports, and indications for use. We carried out a retrospective study utilizing data from the FAERS database, spanning from January 2004 to December 2022. Initially, we downloaded data pertaining to liposomal doxorubicin and conventional doxorubicin (CDOX), which included case ID, primary ID, indications, suspected drugs, adverse events, outcome events, reporter country, reporter type, sex, age, report date, start date, and event date. Following the FDA’s recommendations, we removed duplicate records from the "DEMO" table, retaining only one, and deleted the earliest "FDA_DT" column when the "CASEID" column was identical. We also removed the lesser "PRIMARYID" column when both the "CASEID" and "FDA_DT" columns matched.

### Adverse events and drug identification

MedDRA keywords such as "Anaemia (10002034)", "Back pain (10003988)", "Interstitial lung disease (20000042)", "Pancytopenia (10033661)", "Vomiting (10047700)", among others, were utilized to identify adverse events. Reports involving liposomal doxorubicin and conventional doxorubicin (CDOX) were pinpointed by conducting text string searches for each drug by their generic names, brand names, and abbreviations. We excluded drugs reported in conjunction with the eligible drug listed as interacting or concomitant. DrugBank (The Metabolomics Innovation Centre, Canada, https://go.drugbank.com/) served as a source for batch conversion and compilation of drug names.

### Data mining

Disproportionality analysis and Bayesian analysis comprise four algorithms: the reporting odds ratio (ROR), proportional reporting ratio (PRR), Bayesian confidence propagation neural network (BCPNN), and multi-item gamma poisson shrinker (MGPS). Three of these algorithms were used to establish a connection between liposomal doxorubicin and conventional doxorubicin (CDOX) and adverse events. The equations and standards for these three algorithms were presented in Table 6^23–28,30^. Unless otherwise specified, the current study employs an adjusted disproportionality (adjusted ROR) that accounts for the use of concurrent agents. In addition, the onset time and outcomes of the adverse events associated with liposomal doxorubicin and CDOX were collected and compared.

**Table 6:** Summary of major algorithms used for signal detection.

Algorithms	Equation	Criteria
ROR	ROR = (a/b)/(c/d)	95% CI > 1, N ≥ 2
PRR	PRR = (a/(a + c))/(b/(b + d)); χ^2^ = Σ ((O − E)2/E); (O = a, E = (a + b) (a + c)/(a + b + c + d))	PRR ≥ 2, χ^2^ ≥ 4, N ≥ 3
BCPNN	IC = log2a (a + b + c + d)/((a + c) (a + b))	IC025 > 0

*a* number of reports containing both the suspect drug and the suspect adverse drug reaction, *b* number of reports containing the suspect adverse drug reaction with other medications (except the drug of interest), *c* number of reports containing the suspect drug with other adverse drug reactions (except the event of interest), *d* number of reports containing other medications and other adverse drug reactions. *ROR* reporting odds ratio, *CI* confidence interval, *N* the number of co-occurrences, *PRR* proportional reporting ratio, *BCPNN* Bayesian confidence propagation neural network, *IC* information component, *IC025* the lower limit of the 95% two-sided CI of the IC, 95% CI = eIn (ROR) ± 1.96√(1/a + 1/b + 1/c + 1/d).

### Statistical analysis

The clinical characteristics of patients extracted from the FAERS database were summarized and compared using descriptive analysis. The onset time of adverse events across different drugs was compared using nonparametric tests (Mann–Whitney test for two groups, and the Kruskal–Wallis test for three groups). Fisher’s exact test or Pearson’s chi-square test was employed to compare outcome events across different drugs. Statistical significance was determined with a 95% confidence interval (CI) and p < 0.05. Both data mining and statistical analyses were conducted using Excel and SPSS version 25.0 (IBM Corporation, Armonk, New York, United States).

## Data Availability

The FAERS database utilized in this study was available at: https://fis.fda.gov/extensions/FPD-QDE-FAERS/FPD-QDE-FAERS.html.
